# Stomatin-like protein 2 deficiency results in impaired mitochondrial translation

**DOI:** 10.1371/journal.pone.0179967

**Published:** 2017-06-27

**Authors:** Panagiotis Mitsopoulos, Orsolya Lapohos, Woranontee Weraarpachai, Hana Antonicka, Yu-Han Chang, Joaquín Madrenas

**Affiliations:** 1 Microbiome and Disease Tolerance Centre, Department of Microbiology and Immunology, McGill University, Montreal, Quebec, Canada; 2 Los Angeles Biomedical Research Institute at Harbor-UCLA Medical Center, Torrance, California, United States of America; 3 Department of Human Genetics and Montreal Neurological Institute, McGill University, Montreal, Quebec, Canada; 4 Department of Biochemistry, Faculty of Medicine, Chiang Mai University, Chiang Mai, Thailand; University of Cincinnati College of Medicine, UNITED STATES

## Abstract

Mitochondria translate the RNAs for 13 core polypeptides of respiratory chain and ATP synthase complexes that are essential for the assembly and function of these complexes. This process occurs in close proximity to the mitochondrial inner membrane. However, the mechanisms and molecular machinery involved in mitochondrial translation are not fully understood, and defects in this process can result in severe diseases. Stomatin-like protein (SLP)-2 is a mainly mitochondrial protein that forms cardiolipin- and prohibitin-enriched microdomains in the mitochondrial inner membrane that are important for the formation of respiratory supercomplexes and their function. Given this regulatory role of SLP-2 in processes closely associated with the mitochondrial inner membrane, we hypothesized that the function of SLP-2 would have an impact on mitochondrial translation. ^35^S-Methionine/cysteine pulse labeling of resting or activated T cells from T cell-specific *Slp-2* knockout mice showed a significant impairment in the production of several mitochondrial DNA-encoded polypeptides following T cell activation, including Cytb, COXI, COXII, COXIII, and ATP6. Measurement of mitochondrial DNA stability and mitochondrial transcription revealed that this impairment was at the post-transcriptional level. Examination of mitochondrial ribosome assembly showed that SLP-2 migrated in sucrose-density gradients similarly to the large ribosomal subunit but that its deletion at the genetic level did not affect mitochondrial ribosome assembly. Functionally, the impairment in mitochondrial translation correlated with decreased interleukin-2 production in activated T cells. Altogether, these data show that SLP-2 acts as a general regulator of mitochondrial translation.

## Introduction

Mitochondria are essential for the function of most mammalian cells. These organelles are vital for a host of cellular processes and are typically the chief source of cellular energy production due to their ability to perform oxidative phosphorylation (OXPHOS) [1]. Though most genes encoding mitochondrial proteins are located in the nucleus, mitochondrial (mt) DNA encodes 37 genes of which 13 code for essential polypeptide subunits of respiratory chain complexes I, III, IV, and V, while the remaining two ribosomal (r) RNA and 22 transfer (t) RNA genes are necessary for mitochondrial translation [2, 3]. Defects in the processes leading to expression of mitochondria-encoded polypeptides, including mtDNA maintenance, transcription, and translation, can result in improper assembly and function of mitochondrial respiratory chain complexes, a feature common to a heterogeneous set of severe, often fatal, mitochondrial diseases [4].

Mitochondrial genes are transcribed and translated in the matrix by processes involving unique molecular machinery that is separate and distinct from their nuclear and cytoplasmic counterparts. With regards to translation, mitochondrial ribosomes (mitoribosomes) are made of components generated from both the nuclear and mitochondrial genomes [5]. The assembly of these components is hypothesized to occur in two mitochondrial subcompartments in two steps: early in nucleoids, which are centers of mtDNA maintenance, replication and transcription; and later in RNA granules, locations where post-transcriptional RNA processing and maturation occur [6, 7]. Furthermore, it is known that mitochondrial translation occurs in close proximity to the mitochondrial inner membrane [8, 9]. Indeed, a biochemical study showed that nearly half of mammalian mitoribosomes interact with the mitochondrial inner membrane [10], suggesting that this process is mediated in part by mitoribosome interaction with integral or membrane-bound proteins.

Recent evidence in yeast mitochondria indicates that the mitoribosome binds to membrane-associated proteins [11] and becomes anchored to the inner membrane during the translation of nascent polypeptides [12]. A similar process may occur during mammalian mitochondrial translation such that the 13 extremely hydrophobic core proteins of the respiratory chain are available for immediate membrane insertion during a coordinated process with nuclear-encoded proteins in which assembly of the respiratory chain complexes occurs. In this context, the local microenvironment of the mitochondrial inner membrane including its lipid and protein composition may be critical for the regulation of this process.

We have previously shown that stomatin-like protein (SLP)-2, a mainly mitochondrial protein of the SPFH family of proteins, binds to cardiolipin and functions to form specialized membrane microdomains involving cardiolipin and prohibitins in the mitochondrial inner membrane that are important for the activities of certain respiratory chain complexes [13, 14], and the formation of respiratory chain supercomplexes [15]. Given this regulatory role of SLP-2, it is possible that its function may regulate other processes that depend on compartmentalization of the mitochondrial inner membrane, including translation. Here, we show that SLP-2 is required for *de novo* mitochondrial protein synthesis upon T cell activation in mice, and that this function is not mediated at the level of mtDNA stability or mitochondrial transcription. Furthermore, we show that SLP-2 migrates in sucrose-density gradients similarly to mitoribosome complexes but that its deletion at the genetic level does not alter mitoribosome assembly. Finally, the impairment in mitochondrial translation correlates with decreased T cell activation. These data identify SLP-2 as a key regulator of mitochondrial translation during T cell activation, a function that may extend to other tissues or cell-types.

## Results

### De novo synthesis of mitochondria-encoded polypeptides is dependent on SLP-2 in activated T cells

**Fig 1:**
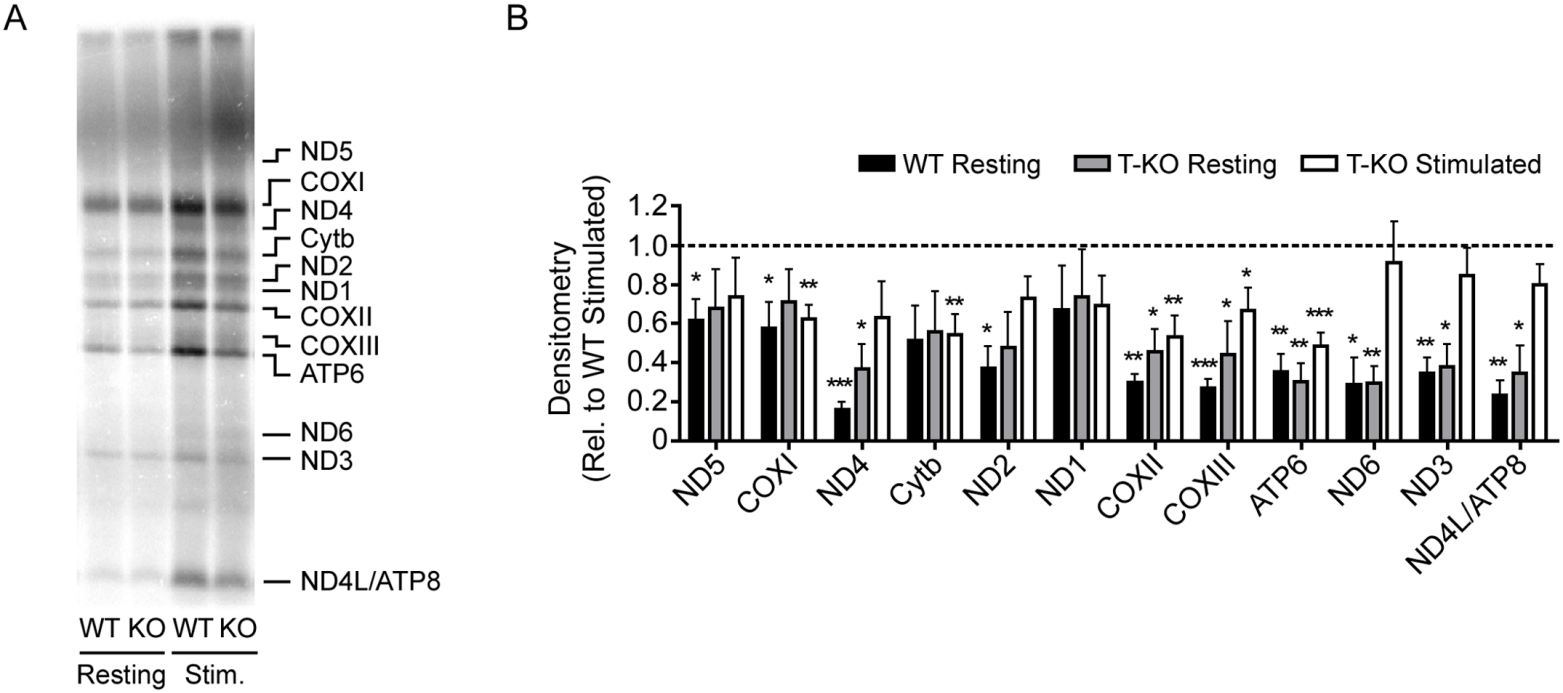
Mitochondrial translation is impaired in SLP-2-deficient T cells upon stimulation. Resting or anti-CD3/CD28-stimulated T cells isolated from WT or SLP-2 T-KO mice were cultured with ^35^S-methionine/cysteine for 1 h in the presence of the cytoplasmic translation inhibitor, emetine. Cell lysates were resolved by SDS-PAGE and ^35^S-methionine/cysteine incorporation into nascent mitochondria-encoded polypeptides was measured by direct autoradiography. (A) Representative ^35^S-methionine/cysteine incorporation data are shown. Bands corresponding to mitochondrial polypeptides are denoted. (B) Densitometric analysis of ^35^S-methionine/cysteine incorporation from multiple experiments was performed with all values relative to the WT Stimulated group, which was assigned a value of 1 (represented by dotted line). Values below dotted line indicate decreased densitometry relative to WT Stimulated group. N = 4 (resting) or 7 (stimulated) mice per group. Statistical significance was calculated using Student's t test. *: p < 0.05; **: p < 0.01; ***: p < 0.001 compared to WT Stimulated group.

To investigate whether SLP-2 functions to directly or indirectly regulate mitochondrial translation, we measured *de novo* mitochondrial protein synthesis in resting or stimulated T cells from wild type (WT) or T cell-specific *Slp-2* knockout (SLP-2 T-KO) mice by detection of ^35^S-methionine/cysteine incorporation into nascent mitochondrial polypeptides (Fig 1A). We found that in WT T cells, the *de novo* level of every mitochondria-encoded polypeptide was lower during resting conditions than upon activation (48 hours post-stimulation), with 11 of 13 polypeptides being significantly less abundant (Fig 1B). Under resting conditions, deletion of *Slp-2* had no effect on mitochondrial translation compared to WT control cells. However, upon stimulation, SLP-2-deficient T cells failed to upregulate the translation of many mitochondria-encoded polypeptides to the same extent as stimulated WT T cells, with *de novo* COXI, Cytb, COXII, COXIII, and ATP6 protein levels being significantly lower than in activated WT T cells. The finding that multiple mitochondria-encoded polypeptides failed to be upregulated in SLP-2-deficient T cells following T cell activation suggested a general defect in mitochondrial translation in the absence of SLP-2.

### The impairment of mitochondria-encoded polypeptide synthesis in SLP-2-deficient T cells occurs post-transcriptionally

**Fig 2:**
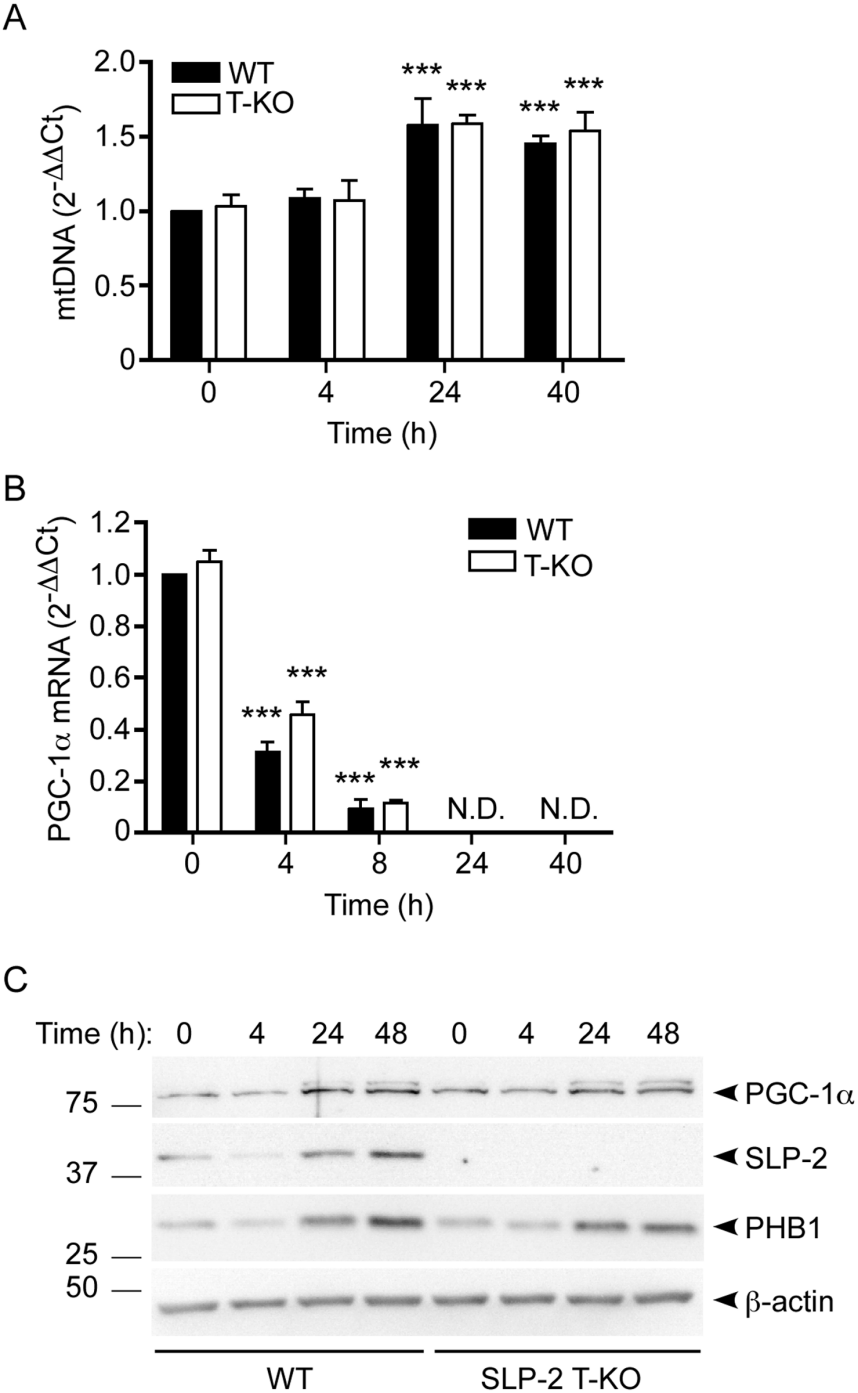
Mitochondrial DNA stability and PGC-1α expression are not affected by deletion of *Slp-2* in T cells. WT and SLP-2 T-KO T cells were resting or stimulated with anti-CD3/CD28 for the indicated times, then total cellular DNA (A), RNA (B) or protein (C) content was isolated. (A) Levels of mtDNA were measured by qPCR and normalized to Rag1 DNA content. Values are relative to the 0 h WT condition. N = 3 mice per group. Statistical significance was calculated using two-way ANOVA. ***: p < 0.001 compared to respective 0 h WT or T-KO control. (B) PGC-1α gene expression was measured by qRT-PCR and normalized to RPL19, B2M, and HPRT gene expression. Values are relative to the 0 h WT condition. N = 4 mice per group. Statistical significance was calculated using two-way ANOVA. ***: p < 0.001 compared to respective 0 h WT or T-KO control. N.D.: Not detected. (C) Cell lysates were resolved by SDS-PAGE, transferred to membranes and immunoblotted with antibodies against PGC-1α, SLP-2, PHB1, and β-actin. Left panel: representative blots are depicted; numbers indicate molecular weight in kDa. The upper band in the anti-PGC-1α blot shows post-translational modification of this protein that may reflect active phosphorylated PGC-1α. Right panel: densitometry was calculated relative to the 0 h WT condition for PGC-1α, SLP-2, and PHB1 (N = 3). Statistical significance was calculated using Student's t test. *: p < 0.05 compared to 0 h WT control. N.D.: Not detected.

General defects in the translation of mitochondria-encoded genes may be secondary to a variety of factors, including poor maintenance of mtDNA, a defect in mitochondrial transcription, or improper assembly of the mitoribosome [2]. To narrow down these possibilities, we first assessed mtDNA stability by measuring its abundance in WT or SLP-2-deficient T cells under resting conditions or during T cell activation by quantitative PCR. Given our previous finding that overexpression of SLP-2 in human T cells increased mtDNA content [13, 16], it was possible that a lack of SLP-2 in mouse T cells would have a negative impact on mtDNA maintenance. We found that both WT and SLP-2-deficient T cells had significantly increased mtDNA content upon T cell activation after 24 and 40 hours when compared to their respective unstimulated controls (Fig 2A). There were no differences in mtDNA levels between WT and SLP-2-deficient T cells at any time studied, indicating that the mitochondrial translation defect in SLP-2-deficient T cells was not related to mtDNA maintenance.

Peroxisome proliferator-activated receptor γ coactivator-1α (PGC-1α) is a master regulator of mitochondrial biogenesis and metabolism [17, 18]. PGC-1α expression is increased in situations of cellular energy stress and can be regulated by various post-translational modifications including phosphorylation of multiple residues [17, 18]. We have previously reported that SLP-2 overexpression increased PGC-1α gene and protein expression in human T cells, and that SLP-2 was upregulated prior to PGC-1α upregulation in control human peripheral blood mononuclear cells following stimulation [13]. To determine whether the disparity in mitochondrial translation between activated WT and SLP-2 T-KO T cells was due to impaired mitochondrial biogenesis in SLP-2-deficient T cells, we measured the gene and protein expression of PGC-1α under resting conditions and upon T cell activation. We found that while PGC-1α mRNA levels decreased following T cell activation (Fig 2B), total PGC-1α protein levels increased under similar conditions, as did post-translationally-modified PGC-1α (Fig 2C) that may reflect active phosphorylated forms of the protein [19]. SLP-2 and PHB1 protein levels were also substantially increased following T cell activation, corroborating previous findings [13]. However, SLP-2 deficiency did not affect PGC-1α abundance at the gene or protein levels, indicating that mitochondrial biogenesis was not a contributing factor to the defective mitochondrial translation observed in SLP-2-deficient T cells upon activation.

**Fig 3:**
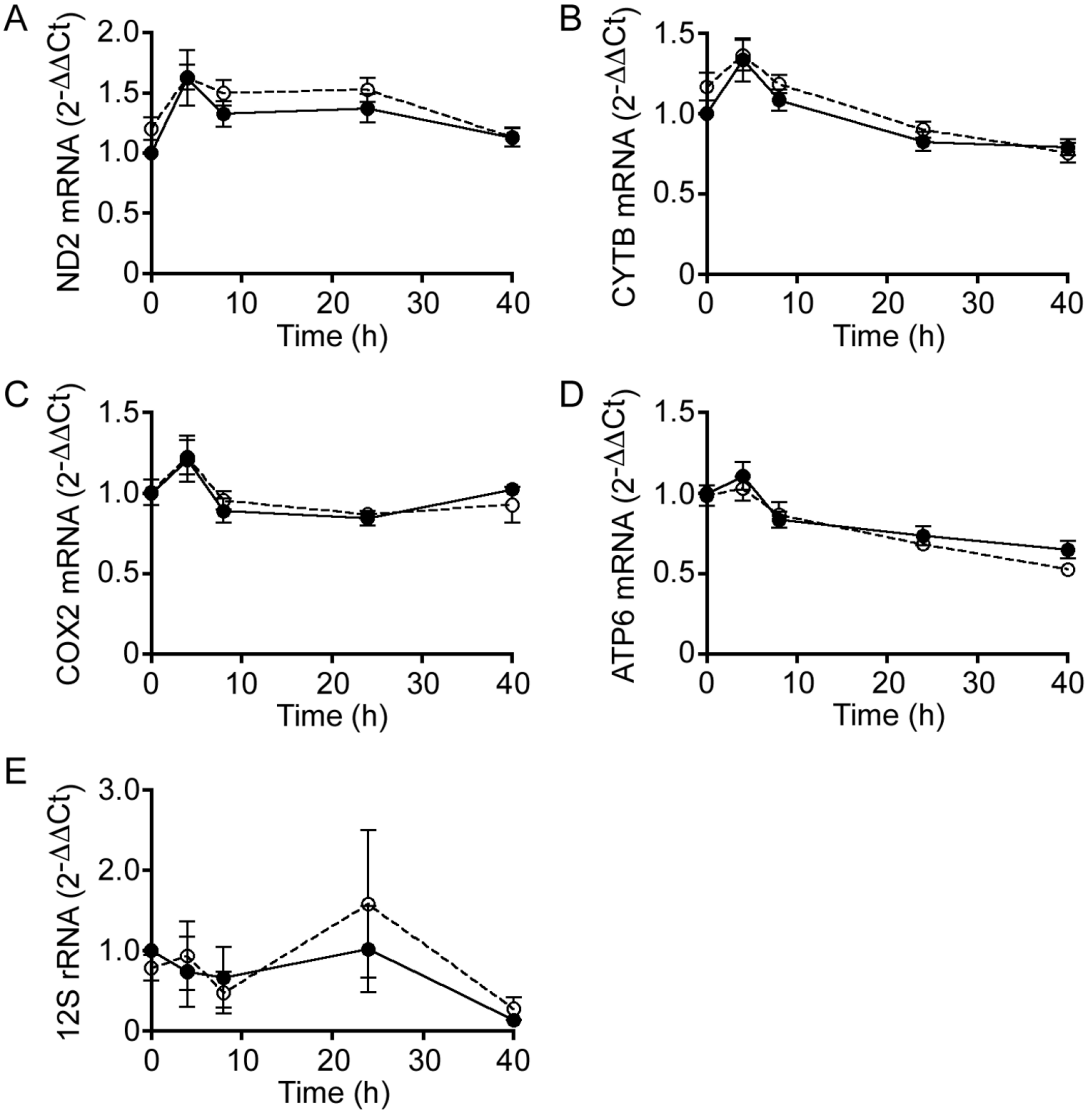
SLP-2 deficiency does not impair mitochondrial transcription in T cells. Total RNA was isolated from resting (i.e., 0 h) or anti-CD3/CD28-stimulated WT (closed circles) or SLP-2 T-KO (open circles) T cells after 4, 8, 24, or 40 h, then qRT-PCR was performed using primers for (A) *Nd2*, (B) *Cytb*, (C) *Cox2*, (D) *Atp6*, and (E) 12S rRNA. Normalization of gene expression was performed using *Rpl19*, *B2m*, and *Hprt*. N = 4 mice per group.

Since there were no apparent defects in mtDNA stability or PGC-1α expression in resting or activated SLP-2-deficient T cells, we next examined whether mitochondrial transcription was occurring normally under these conditions. To test this, we performed quantitative reverse-transcriptase (qRT)-PCR to measure transcript levels of the genes corresponding to four of the most significantly decreased polypeptides, ND2, CYTB, COX2, and ATP6, as shown in Fig 1. Additionally, it should be noted that one gene from each respiratory chain complex is represented in this selection. We found that the transcript levels were equally abundant for each gene both prior to and following T cell activation, and that deletion of *Slp-2* did not affect the abundance of these transcripts compared to WT T cells over time (Fig 3A–3D). Furthermore, the levels of 12S rRNA, an indicator of mitoribosome abundance, were not significantly affected by *Slp-2* deletion (Fig 3E). These results indicated that the mitochondrial translation defect we observed in activated SLP-2-deficient T cells was post-transcriptional.

### SLP-2 migrates in sucrose-density gradients similarly to mitoribosome complexes but does not affect their assembly

**Fig 4:**
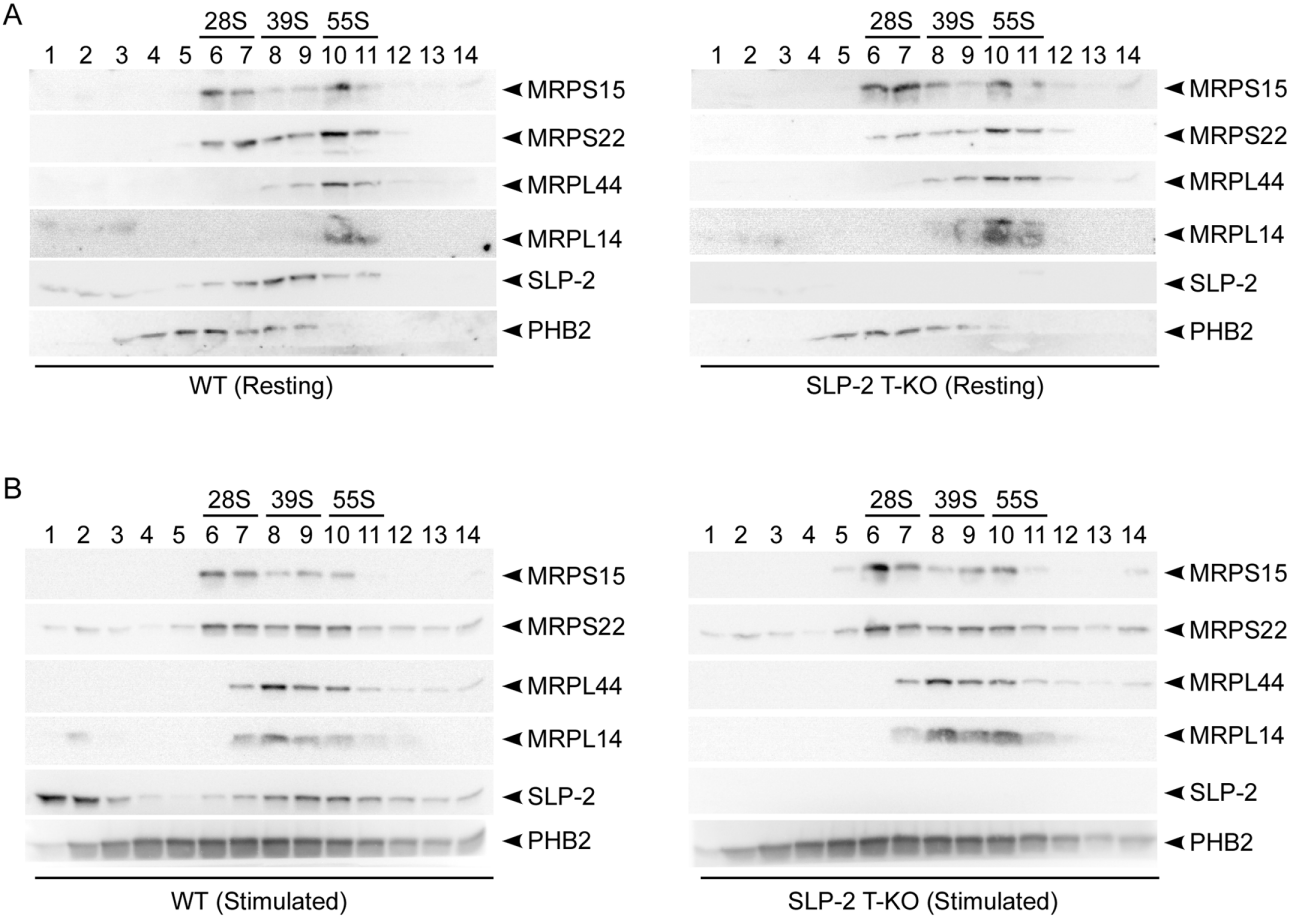
SLP-2 migrates in sucrose-density gradients similarly to mitoribosome complexes but does not affect mitoribosome assembly. Mitochondrial lysates from (A) resting or (B) anti-CD3/CD28-stimulated WT or SLP-2 T-KO T cells were layered onto 10–30% discontinuous sucrose gradients. Following sedimentation, 14 equal fractions were collected with fraction 1 starting from the top (10% sucrose) to fraction 14 at the bottom (30% sucrose) of the column. The total protein content of each fraction was resolved by SDS-PAGE then transferred to membranes that were blotted against anti-MRPS15, anti-MRPS22, anti-MRPL44, anti-MRPL14, anti-SLP-2, and anti-PHB2 antibodies. Fractions corresponding to the small (i.e., 28S) and large (i.e., 39S) mitoribosomal subunits, as well as the assembled mitoribosome (i.e., 55S) are indicated. Fraction numbers are indicated for each set of blots. Data are representative of two (A) or three (B) independent experiments.

The assembly and stability of the mitoribosome is critical for mitochondrial translation to occur. Mammalian mitoribosomes are composed of a 28S small and 39S large subunit to form a 55S monosome [20]. The small subunit is composed of 12S rRNA and approximately 30 mitoribosomal proteins (MRPs), whereas the large subunit is composed of 16S rRNA and approximately 53 MRPs [2]. To assess mitoribosome assembly in the absence of SLP-2, we separated mitochondrial lysates of resting or stimulated WT or SLP-2 T-KO T cells based on size by sucrose density sedimentation, then collected and resolved 14 equal fractions by SDS-PAGE. Immunoblots of protein components of the small (e.g., MRPS15, MRPS22) or large (e.g., MRPL14, MRPL44) mitoribosomal subunits revealed that under resting conditions, the amounts of the small and large mitoribosomal subunits, as well as amounts of fully assembled monosomes were not different in SLP-2-deficient T cells compared to WT T cells (Fig 4A). Stimulated SLP-2 T-KO T cells exhibited a similar lack of differences in mitoribosome assembly as compared to WT T cells under the same conditions (Fig 4B). Interestingly, although some SLP-2 remained in less dense fractions at the top of the gradient, this protein mainly sedimented across fractions that contained mitoribosomal subunits in both resting and stimulated conditions. In fact, upon stimulation the sedimentation profile of SLP-2 shifted into denser fractions and was more abundant in those containing 55S monosomes compared to resting cells. The overlap in these sedimentation profiles indicates that we cannot exclude the possibility of a direct or indirect interaction between mitoribosomes and SLP-2. Immunoblotting of PHB2 revealed that its sedimentation profile did not match that of the mitoribosomes indicating that they are not directly associated. Altogether, these data document an important post-transcriptional function for SLP-2 in the production of mitochondria-encoded polypeptides.

### The impaired synthesis of mitochondria-encoded polypeptides in the absence of SLP-2 correlates with decreased T cell activation

**Fig 5:**
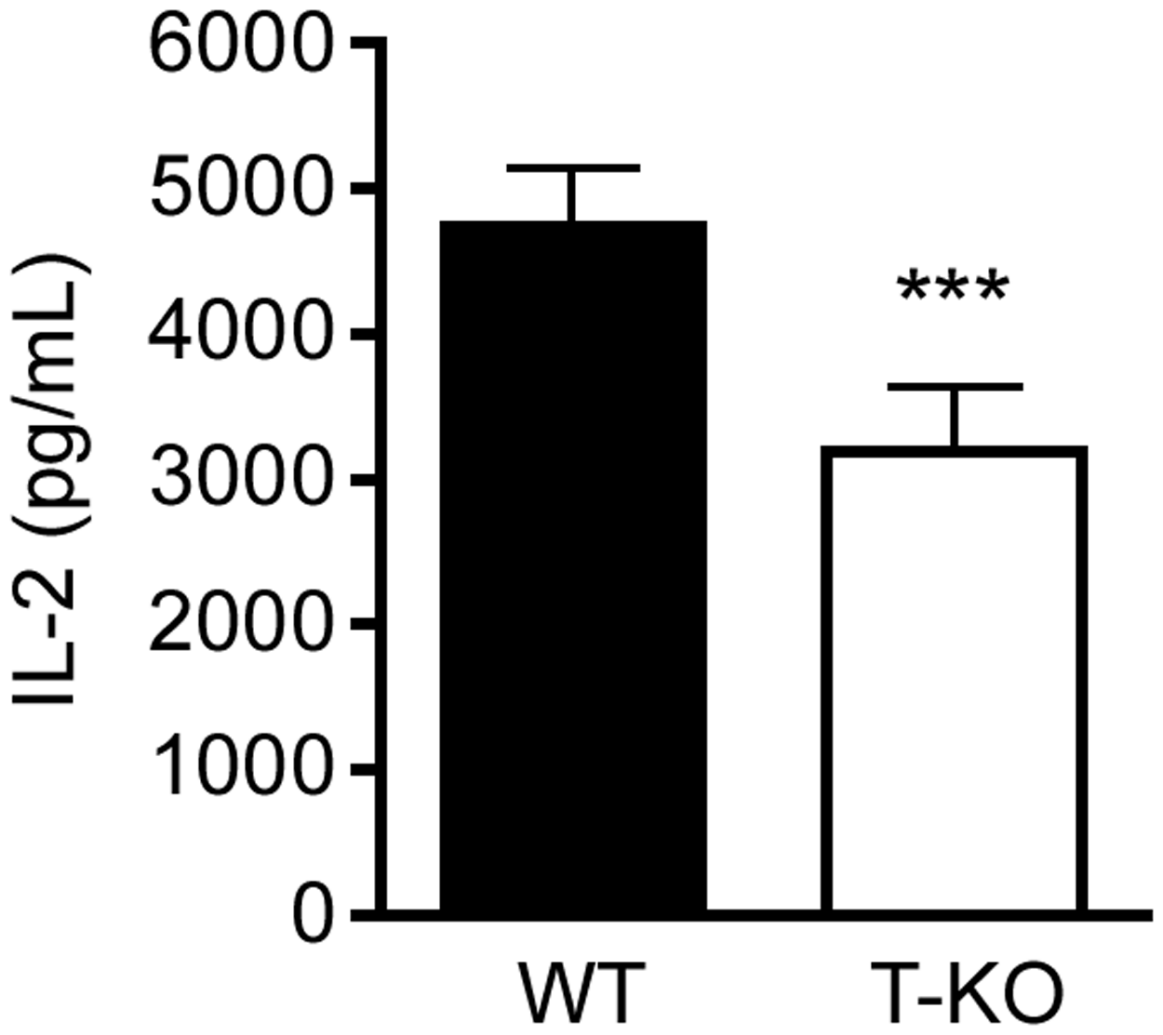
IL-2 production is decreased in SLP-2 T-KO T cells following activation. Splenic T cells were isolated from WT or SLP-2 T-KO mice and stimulated with anti-CD3/CD28. Secretion of IL-2 was measured after 18 h by ELISA. N = 5 mice per group. Statistical significance was calculated using Student's t test. ***: p < 0.001 compared to WT group.

To determine whether the defect in mitochondrial translation occurring in activated SLP-2-deficient T cells was functionally relevant, we measured interleukin (IL)-2 production in WT and SLP-2 T-KO T cells stimulated through the antigen receptor. T cell activation is a vital process in the adaptive immune response that is required for T cell differentiation and proliferation [21]. In accordance with our previous findings [14, 15], we found that T cells lacking SLP-2 had significantly decreased IL-2 production compared to their WT counterparts (Fig 5). These data indicate that the defect in mitochondrial translation occurring in the absence of SLP-2 has significant consequences on the function of T cells.

## Discussion

The mechanisms and molecular machinery required for the translation of mitochondria-encoded genes are not fully defined. Our findings identify SLP-2 as a vital protein for efficient mitochondrial translation during the metabolically demanding conditions accompanying T cell activation. Indeed, our data indicate that SLP-2 is important as a general regulator of mitochondrial translation since the expression of multiple polypeptides, namely Cytb, COXI, COXII, COXIII, and ATP6, is significantly affected by its absence. This defect in mitochondrial translation correlates with a functional impairment in T cell activation. Though the mechanism underlying this function of SLP-2 remains uncertain, the data presented here narrow the focus to a post-transcriptional role.

Deletion of *Slp-2* did not affect mitoribosome assembly, although we found that SLP-2 migrated in sucrose-density gradients similarly to the large mitoribosomal subunit and to a lesser extent the assembled monosome. This finding is corroborated by a study showing SLP-2 co-migration with the 39S mitoribosome complex by complexome profiling [22]. More importantly, compared to resting conditions our data showed that SLP-2 migration increased in fractions containing the monosome during T cell activation, correlating with impaired translation efficiency of mitochondria-encoded polypeptides in SLP-2-deficient cells. Furthermore, deletion of *Slp-2* did not affect mitoribosome abundance. These findings indicate that SLP-2 is either directly or indirectly involved in the function of the mitoribosome and affects its efficiency, but by itself it is not essential for its assembly since the mitoribosome migration profiles were unchanged upon *Slp-2* deletion.

Mitochondrial translation is closely associated with the mitochondrial inner membrane, but its functional role in this process is poorly understood. Since SLP-2 forms specialized prohibitin- and cardiolipin-enriched microdomains in the mitochondrial inner membrane that are associated with the activities of certain respiratory complexes and the formation of respiratory chain supercomplexes, it is likely that SLP-2 may regulate mitochondrial translation by controlling the membrane composition at sites where translation occurs. We examined the migration profile of PHB2 with respect to mitoribosome complexes and found that its sedimentation profile on the sucrose gradient did not match that of mitoribosomes. However, a small amount of PHB2 was present in the same fractions as the small mitoribosomal subunit, in agreement with previous studies reporting that prohibitins contributed to mitochondrial translation [23] and co-migrated with the 28S subunit [22]. Taken together, the sucrose gradient sedimentation pattern suggests a weak or indirect association of prohibitins with the mitoribosome. Interestingly however, though the absence of SLP-2 impaired mitochondrial translation in activated T cells, both mitoribosome assembly and the migration of PHB2 were unchanged relative to activated WT T cells. This implies that SLP-2 is not by itself responsible for monosome assembly or the potential association of prohibitins with the mitoribosome.

Finally, we found that although T cells lacking SLP-2 were able to produce each mitochondria-encoded polypeptide, efficient mitochondrial translation was dependent on SLP-2 only following T cell activation. Initially this seemed counter-intuitive since OXPHOS is the predominant form of energy production in resting T cells and is supplanted by aerobic glycolysis following T cell activation [24]. However, not only is OXPHOS still engaged in activated T cells [15], it is significantly increased [25] and is required for the expression of early activation markers and for optimal T cell proliferation [15, 26]. Therefore, it is possible that resting T cells lacking SLP-2 had the opportunity to produce sufficient amounts of mitochondria-encoded polypeptides since they were in a steady state with relatively low metabolic demands. However, activated T cells undergo rapid growth and become metabolically very active to support their proliferation and to exert their immune function [24]. We see this reflected in the significantly increased abundance of each mitochondria-encoded polypeptide in WT T cells during the transition from resting to activated states. However, SLP-2-deficient T cells did not have the capacity to increase mitochondrial translation, highlighting the importance of this protein. Furthermore, this impairment in mitochondrial translation may be a contributing factor to the defective functional responses of SLP-2-deficient T cells observed here and previously, which include decreased IL-2 production and delayed proliferation [15].

In summary, we have identified SLP-2 as a key protein for general mitochondrial translation in activated T cells. Although the mechanism behind this function requires further study, we have shown that SLP-2 migrates in sucrose-density gradients similarly to mitoribosome complexes and hypothesize that it may exert its effect through the compartmentalization of the mitochondrial inner membrane at sites where translation occurs. Finally, we propose that this function may extend to other tissues or cell types, particularly those with great metabolic demands.

## Materials and methods

*Mice*. We generated T cell-specific *Slp-2* knockout mice in the C57BL/6N Tac background, as described previously [14]. Briefly, *Slp-2*^lox/wt^ mice were crossed with CD4-Cre mice (Taconic Farms, Hudson, NY) to generate *Slp-2*^lox/lox^/Cre^+^ (SLP-2 T-KO) or *Slp-2*^lox/lox^/Cre^-^ (WT) mice. Mice were maintained in the animal facility at McGill University with approval of the Comparative Medicine and Animal Resources Centre in accordance with the Canadian Council on Animal Care Guidelines.

*Cell Culture*. T cells were isolated from spleens of WT or SLP-2 T-KO mice. Briefly, spleens were homogenized using physical disruption in cell culture media to obtain a single-cell suspension that was subsequently passed through a 40 μm mesh filter. Red blood cell lysis buffer (144 mM ammonium chloride, 17 mM Tris, pH 7.2) was added for 2 min on ice, then cells were washed three times with cell culture media. T cells were isolated from these splenocytes by negative selection using the EasySep^™^ Mouse T cell Isolation kit (Stemcell Technologies, Vancouver, Canada). Cells were resting or stimulated with 5 μg/mL anti-CD3ε and 2 μg/mL anti-CD28 plate-bound antibodies (eBioscience, San Diego, CA), and cultured in RPMI supplemented with 10% fetal bovine serum, 1 mM sodium pyruvate, 2 mM L-glutamine, 10 mM HEPES, 0.1% β-mercaptoethanol, and penicillin/streptomycin, unless otherwise indicated. Cells were incubated at 37°C in a humidified atmosphere of 5% CO_2_.

*Mitochondrial translation*. Mitochondrial translation was performed as described [27]. Briefly, T cells from WT or SLP-2 T-KO mice were resting or stimulated with anti-CD3/CD28 for 48 h, then washed with PBS and incubated for 30 minutes in labeling media (i.e., methionine/cysteine-free RPMI (Sigma-Aldrich, St. Louis, MO), 10% dialyzed fetal bovine serum (Gibco, Carlsbad, CA), 1 mM sodium pyruvate, 2 mM L-glutamine, 10 mM HEPES, and penicillin/streptomycin). Emetine, a cytoplasmic translation inhibitor, was added to a final concentration of 100 μg/mL. Following incubation for 5 minutes, cells were pulse-labeled with 200 μCi/mL of ^35^S-methionine/cysteine mix (Perkin Elmer, Waltham, MA) and incubated for 1 hour, then media was replaced with R10 + 10% FBS media for 10 minutes. After three washes, cells were resuspended in PBS and frozen at -80°C. Protein levels were measured using the Micro BCA kit (Thermo Fisher Scientific, Waltham, MA). Samples were suspended in gel loading buffer (93 mM Tris-HCl, 7.5% glycerol, 1% SDS, 0.25 mg/mL bromophenol blue, 3% β-mercaptoethanol, pH 6.7), sonicated at 60% for 8 seconds, then 50 μg of each sample was loaded onto a 12–20% gradient polyacrylamide gel and electrophoresed at 8 mA for 16 hours. Gels were dried at 60°C under vacuum for 1 hour using an SGD2000 Slab Gel Dryer (Thermo Fisher Scientific), then exposed to a phosphoimager cassette for 3 days. Labeled translation products were detected by direct autoradiography using a Storm 840 Gel and Blot Imaging System and densitometric analysis was performed using ImageQuant TL software (GE Lifesciences, Buckinghamshire, England).

*Mitoribosome assembly*. All sample processing was performed on ice unless otherwise indicated. Mitochondria were isolated as described [28], with minor modification. Briefly, resting or anti-CD3/CD28-stimulated WT or SLP-2 T-KO T cells were suspended in isolation buffer (320 mM sucrose, 10 mM Tris-HCl, EDTA-free complete protease inhibitor cocktail (Roche, Basel, Switzerland), pH 7.4) and mitochondria were isolated using 50 strokes of a tight-fitting Dounce homogenizer. Cellular debris were pelleted (600 × g, 10 min, 4°C) and discarded. The mitochondria-containing supernatant fraction was then centrifuged (10,000 × g, 10 min, 4°C), then mitochondria were washed with isolation buffer and resuspended at 10 mg/mL in lysis buffer (1% Triton X-100, 260 mM sucrose, 100 mM KCl, 20 mM MgCl_2_, 10 mM Tris-HCl, 5 mM β-mercaptoethanol, EDTA-free complete protease inhibitor cocktail (Roche), pH 7.5) for 20 minutes. Following centrifugation at 9300 × g for 45 min at 4°C, 272 μg of mitochondrial lysates were layered onto cold, 1 mL, 10–30% discontinuous sucrose gradients, then centrifuged at 100,000 × g for 2 h at 4°C using an SW 60 Ti rotor on an Optima LE-80K ultracentrifuge (Beckman Coulter, Indianapolis, IN). Sucrose gradient buffer consisted of lysis buffer lacking Triton X-100. Fourteen fractions of equal volume were collected starting from the top and total protein content was precipitated by addition of 20% trichloroacetic acid. Following three washes with acetone, precipitated proteins were dissolved at room temperature in sample buffer (2% SDS, 2% β-mercaptoethanol, 62.5 mM Tris, pH 6.8, 10% glycerol, 0.5% bromophenol blue), then boiled and resolved by SDS-PAGE. Proteins were transferred to PVDF membranes and then blotted using anti-MRPL14 (Sigma-Aldrich), anti-MRPL44, anti-MRPS15, anti-MRPS22, anti-SLP-2 (Proteintech Group, Rosemont, IL), and anti-PHB2 (Santa Cruz Biotechnology, Santa Cruz, CA) antibodies. Cells from 4 mice per group were pooled to achieve sufficient protein content for detection in each experiment.

*Quantitative PCR*. For quantification of mitochondrial mRNA, total cellular RNA was isolated from resting or anti-CD3/CD28-stimulated WT or SLP-2 T-KO T cells at the indicated times using the RNeasy^®^ Plus Mini kit (Qiagen, Mississauga, ON, Canada). RNA was quantified using NanoDrop (Thermo Scientific, Wilmington, DE), and cDNA was synthesized from 88.3 ng of total RNA using the Omniscript^®^ Reverse Transcription Kit (Qiagen). Transcript levels were quantified on the CFX96^™^ Real-Time System using iTaq^™^ Universal SYBR^®^ Green Supermix (Bio-Rad, Hercules, CA). Primers: ND2 (Fwd.: 5′-CACCCTTGCCATCATCTAC-3′, Rev.: 5′-CTGAATTCCAGGCCTACTC-3′); Cytb (Fwd.: 5′-GTAGACAAAGCCACCTTGAC -3′, Rev.: 5′-CCTGTTGGGTTGTTTGATCC-3′); COX2 (Fwd.: 5′-CCGAGTCGTTCTGCCAATAG-3′, Rev.: 5′-GATTTAGTCGGCCTGGGATG-3′); ATP6 (Fwd.: 5′-ATTAGCCCACCAACAGCTAC-3′, Rev.: 5′-GGCTTACTAGGAGGGTGAATAC-3′); 12S rRNA (Fwd.: 5′- CTGCTCGCCAGAACACTACG -3′, Rev.: 5′- TGAGCAAGAGGTGGTGAGGT -3′).

For mtDNA quantification, WT or SLP-2 T-KO T cells were stimulated for the indicated times with Mouse T Activator Dynabeads (Life Technologies, Grand Island, NY), then washed with PBS and DNA was isolated using the GeneJET Genomic DNA Purification kit and normalized using NanoDrop (Thermo Scientific). The mtDNA primers used (Fwd.: 5′-CTAGAAACCCCGAAACCAAA-3′, Rev.: 5′-CCAGCTATCACCAAGCTCGT-3′) were shown to provide accurate quantification of mtDNA in mouse tissues without co-amplification of nuclear mitochondrial insertion sequences, a confounding factor of accurate mtDNA quantification [29].

*Immunoblotting*. For PGC-1α detection, WT or SLP-2 T-KO T cells were stimulated with Mouse T Activator Dynabeads (Life Technologies) for the indicated times, then washed with ice-cold PBS containing 400 μM sodium orthovanadate and resuspended in lysis buffer containing 1% Triton X-100 with phosphatase and protease inhibitors for 45 min on ice, as described [14]. Lysates were resolved by SDS PAGE and transferred to PVDF membranes, then immunoblotted with antibodies against PGC-1α, PHB1 (Santa Cruz Biotechnology), SLP-2 (Proteintech Group), and β-actin (Cell Signaling Technology, Danvers, MA). Densitometric analysis was performed using FluorChem software (Alpha Innotech, San Jose, CA).

*Enzyme-linked immunosorbent assay (ELISA)*. T cells were isolated from spleens of WT or SLP-2 T-KO mice and stimulated with 5 μg/mL anti-CD3ε and 2 μg/mL anti-CD28 for 18 h. Secretion of IL-2 was measured by ELISA using the mouse IL-2 ELISA kit (eBioscience) according to the manufacturer's instructions.
